# The Association Between Hypertension and Cognitive Impairment, and the Role of Antihypertensive Medications: A Literature Review

**DOI:** 10.7759/cureus.12035

**Published:** 2020-12-11

**Authors:** Nupur Mishra, Devyani Mohan, Sehrish Fuad, Deepak M Basavanagowda, Zaid A Alrashid, Arveen Kaur, Bindu Rathod, Sadia Nosher, Stacey E Heindl

**Affiliations:** 1 Medicine, California Institute of Behavioral Neurosciences & Psychology, Fairfield, USA; 2 Surgery, California Institute of Behavioral Neurosciences & Psychology, Fairfield, USA; 3 Psychiatry and Behavioral Sciences, California Institute of Behavioral Neurosciences & Psychology, Fairfield, USA; 4 Neurology, California Institute of Behavioral Neurosciences & Psychology, Fairfield, USA; 5 Family Medicine, California Institute of Behavioral Neurosciences & Psychology, Fairfield, USA; 6 Medicine, Avalon University School of Medicine, Willemstad, CUW

**Keywords:** angiotensin receptor blockers, ace inhibitors, antihypertensive medications, hypertension, cognitive impairment, thiazide diuretics, calcium channel blockers, cognition, dementia, blood pressure

## Abstract

Vascular dementia (VD) is one of the leading causes of dementia, and hypertension is a known risk factor for VD. Hypertension treatment guidelines have previously discussed an optimal blood pressure goal to prevent further cardiovascular complications with long-term management. The treatment of hypertension can prevent stroke, kidney failure, and perhaps prevent cognitive decline as well. We reviewed studies that demonstrated an association between hypertension and cognitive impairment (CI). The role of antihypertensive medications (AHM) in preventing CI was also investigated. This topic is worth exploring as dementia has high healthcare costs and will become prominent as the population in the United States ages. We used the medical subject heading (MeSH) search strategy on Pubmed and reviewed 22 articles. The studies showed that there might be a link between hypertension, AHM, and CI. The studies did not suggest a superiority of any specific AHM class to prevent CI. Further research on optimal hypertension treatment goals to prevent cognitive impairment and dementia is recommended.

## Introduction and background

Hypertension guidelines have been a controversial topic in recent years [1]. The 2017 ACC/AHA guideline for the prevention, detection, evaluation, and management of high blood pressure in adults (2017 ACC/AHA guidelines) defined hypertension as any systolic blood pressure (SBP) greater than 130 mmHg (≥130 mmHg), or diastolic blood pressure (DBP) greater than 80 mmHg (≥80 mmHg) [2]. These guidelines were changed to incorporate blood pressure (BP) in a spectrum as being normal (SBP <120 and DBP <80 mmHg), elevated (SBP 120-129 and DBP <80 mmHg), stage one high BP (SBP 130-139 mmHg or DBP 80-89 mmHg), and stage two high BP (SBP ≥140 or DBP ≥90 mmHg). The 2017 ACC/AHA guidelines suggested using a treatment goal of ≤130/80 mmHg for all ages, and all comorbidities, with few exceptions [2].

This treatment goal differs from the earlier 2014 Eighth Joint National Committee (JNC-VIII) guidelines for hypertension. Their findings suggested that in ages 60 or greater, a treatment goal of ≤150/90 mmHg should be pursued with pharmacologic treatment. For ages 30 to 59, they suggested a treatment goal of ≤140/90 mmHg, with pharmacologic therapy [3]. Apart from referring to age, the JNC-VIII guidelines also established chronic kidney disease (CKD) and diabetes as important comorbidities and separated this patient population from the rest of the general patient population. They specifically mentioned patients with cardiovascular diseases such as stroke as high-risk populations, and the panel members could not reach unanimity for SBP treatment goals in these high-risk groups [3].

**Figure 1:**
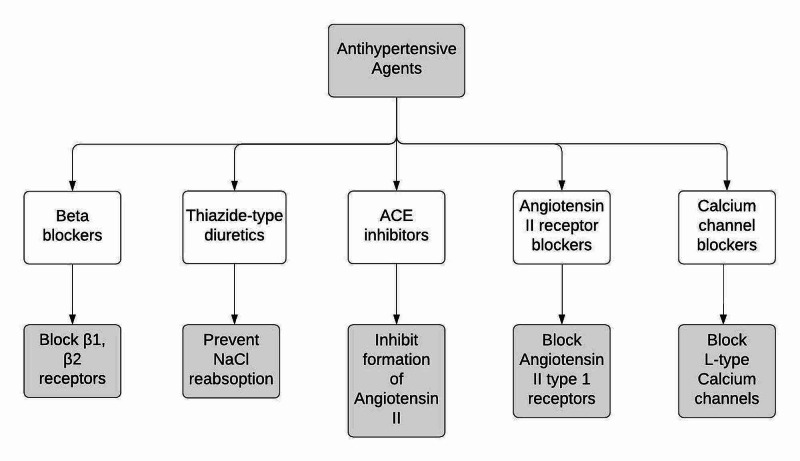
Classification of antihypertensive medications and their mechanism NaCl: sodium chloride, ACE: angiotensin-converting enzyme

In terms of treatment, the 2014 JNC-VIII guidelines suggested diabetes, CKD, and race as a specific patient population group with specific antihypertensive medication guidelines [3]. As such, the treatment algorithm from JNC-VIII guidelines suggested the use of angiotensin-converting enzyme inhibitors (ACEi) or angiotensin receptor blockers (ARB) for hypertensive patients with CKD for all races, thiazides or calcium channel blockers (CCB) for black, non-CKD hypertensive patients, and ACE/ARB, thiazides, or CCB for non-black, non-CKD hypertensive patients (Figure 1) [3].

The 2017 ACC/AHA guidelines suggested that relative risk reduction for cardiovascular diseases (specifically noted as chronic heart disease, heart failure, and stroke) by using antihypertensive medications (AHM) was fairly similar across the general patient population in terms of age, sex, body mass, CKD, diabetes mellitus (DM), and atrial fibrillation (AF) [2]. They recommended starting pharmacologic treatment for anyone with stage two high BP and anyone with stage one high BP plus a clinical atherosclerotic cardiovascular disease (ASCVD) risk of ≥10%. For treatment strategy, the study indicated leniency to chose any antihypertensive, except in certain cases such as race or clinical comorbidity. For example, in cases where a far greater benefit was observed, i.e., beta-blockers (BB) after myocardial infarction, diuretics in heart failure patients (Figure 1).

These guidelines also highlighted the impact of cardiovascular diseases, such as chronic heart disease, myocardial infarction, stroke [2], and suggested treating BP to prevent the development of cognitive decline (CD) and dementia [2,4-9]. Chronic high blood pressure is also a risk factor for cerebrovascular disease, leading to vascular dementia complications. The prevalence of hypertension seemed to increase with age and was greatest in those more than 75 years of age, with >79% of this population being hypertensive [2]. The elderly patient population also reported the greatest cognitive dysfunction, including the spectrum from mild cognitive impairment (MCI) to dementia [10,11]

Hypertension might be a contributing risk factor for MCI and dementia and can be targeted for therapy [12-14]. The healthcare burden of taking care of elderly patients with dementia is not a small number, and Hurd et al. even placed the cost to be among the highest healthcare expenditure, including heart disease and cancer [15]. Findings from observational studies suggested that treating hypertension in midlife and long-term might prevent CD in the elderly patient population [2,14,16]. However, treating hypertension in the elderly population and its contributing effect on cognition is not very clear. The question is, how and why does hypertension affect cognition, and can AHM play a role in preventing cognitive dysfunction?

Cognitive dysfunction covers a spectrum from MCI to dementia [12,17]. The pathophysiology surrounding cognitive dysfunction is varied. Some etiologies of dementia, such as Alzheimer’s disease (AD), have novel treatments; however, there is no specific treatment for vascular dementia [12]. Over time, as the United States population ages, it is becoming imperative that we have a greater understanding of treating hypertension in elderly populations. The topic is worth investigating and few studies have also developed study protocols and feasibility trials for studying vascular dementia [18,19]. In keeping with these views, the purpose of this review was to investigate studies that dealt with hypertension and AHM and to assess their association with cognitive dysfunction.

## Review

Methods

Search Strategy

We searched PubMed and MeSH search strategy to find as many as possible relevant articles. The keywords included are listed in Tables 1, 2. After manually screening for duplicate and unavailable studies, articles were also screened for their relevance to the topic. A total of 22 articles were identified for discussion.

**Table 1:** PubMed keywords that dealt with specific antihypertensive medications and cognition ACE: angiotensin-converting enzyme

PubMed keywords	Database	Number of studies	Number of studies with criteria applied	Number after removing irrelevant, unavailable, and duplicate studies
Thiazide + cognitive	PubMed	138	1	1
Calcium channel blocker + cognitive	PubMed	1217	31	6
Angiotensin receptor blocker + cognitive	PubMed	314	16	1
ACE inhibitors + cognitive	PubMed	447	13	0

**Table 2:** MeSH keywords that dealt with hypertension, antihypertensive medications, blood pressure, and cognitive dysfunction MeSH: medical subject heading

MeSH keywords	Database	Number of studies	Number of studies with criteria applied	The number after removing irrelevant, unavailable, and duplicate studies
Hypertension + cognitive dysfunction	PubMed	260	14	6
Antihypertensive agents + cognitive dysfunction	PubMed	71	12	4
Blood pressure + cognitive dysfunction	PubMed	158	17	4

Eligibility Criteria

One author screened the available abstracts using the following inclusion/exclusion criteria. We included studies within the past five years (2015-2020) to review the latest available data. Only papers in the English language were selected. We excluded all studies except clinical trials, meta-analysis, and randomized controlled trials. One animal study was also included. All ages and gender of patients were included. All geographical locations were also included.

Results

Study Selection and Characteristics

Studies collected from the methods were sorted based on relevance to subtopics and included in Tables 3-6. A total of eleven meta-analyses, four clinical trials, and seven randomized clinical trials (RCT) were included.

**Table 3:** Studies that showed a link between cognition and cardiovascular risk factors APOEe4: apolipoprotein E4, MCI: mild cognitive impairment, ACEi: angiotensin-converting enzyme inhibitor, MI: myocardial infarction, CCB: calcium channel blockers, CI: cognitive impairment, BP: blood pressure, POCD: postoperative cognitive decline

Authors	Year	Type of study	Total number of patients	Relevant results and conclusions
Xue et al. [20]	2019	Meta-analysis	6829	Not having an APOEe4 allele, or no hypertension, or no history of stroke were associated with a greater chance to revert from MCI to normal cognition
Xie et al. [21]	2018	Meta-analysis	143,095	ACEi has significantly reduced all secondary outcomes (all-cause mortality, MI, and stroke). CCB and diuretics reduced stroke significantly.
You et al. [22]	2017	Randomized clinical trial	231	Frequency of CI after Intracerebral hemorrhage may be linked to age, gender, initial neurological deficit, and high early systolic BP in the first 24 hours.
Sun et al. [23]	2016	Randomized clinical trial	340	Intraoperative use of nicardipine and nitroglycerin along with esmolol may reduce the incidence of POCD
Scharf et al. [24]	2019	Clinical trial	554	Age, hypertension, and fasting glucose in males predicted the progression of white matter hyperintensity.
de Heus et al. [25]	2019	Clinical trial	460	Greater blood pressure variability might be associated with the progression of Alzheimer disease
Georgakis et al. [26]	2017	Meta-analysis, systematic review	38,257	Left ventricular hypertrophy might be independently linked with an increased chance of CI

**Table 4:** Studies that involved the use of nonspecific antihypertensive medications and cognition CI: cognitive impairment, SBP: systolic blood pressure, MCI: mild cognitive impairment

Authors	Year	Type of study	Total number of patients	Relevant results and conclusions
Parsons et al. [27]	2016	Meta-analysis	57,049	Use of antihypertensive medications significantly lowered the risk of stroke, but not dementia or cognitive decline
Hughes et al. [28]	2020	Meta-analysis, systematic review	96,158	Use of antihypertensive medications significantly lowered the risk of dementia or CI when compared to control
Xu et al. [29]	2017	Meta-analysis	30,895	Use of antihypertensive medications was associated with reduced risk of dementia
SPRINT MIND Investigators for the SPRINT Research Group et al. [12]	2019	Randomized clinical trial	9,361	Treatment of SBP goal to less than 120 mmHg did not improve the risk of dementia than the SBP goal to less than 140 mmHg, but it did improve the risk of MCI.
Vazirinejad et al. [30]	2019	Clinical trial	248	Use of antihypertensive medications in ages 40 years or greater showed an improved level of cognition performance
Moonen et al. [31]	2015	Randomized clinical trial	385	Stopping antihypertensive medications in elderly patients with MCI did not improve cognitive, psychological, or daily functioning

**Table 5:** Studies that looked at the association between specific antihypertensive medications and cognition CBF: cerebral blood flow, AD: Alzheimer’s disease, RAS: renin-angiotensin system, AHM: antihypertensive medications Note: One animal study included [35].

Authors	Year	Type of study	Total number of cases	Relevant results and conclusions
DIURETICS				
Tully et al. [32]	2016	Meta-analysis, systematic review	52,599	The use of diuretic antihypertensive medications might reduce the risk of dementia
CALCIUM CHANNEL BLOCKERS				
Lawlor et al. [33]	2018	Clinical trial	511	Nilvadipine might not be useful in older aged patients with AD
de Jong et al. [34]	2019	Randomized clinical trial	58	Nilvadipine increased CBF in the hippocampus and has positive cerebrovascular effects in AD.
ANGIOTENSIN II RECEPTOR BLOCKERS				
Ahmed et al. [35]	2018	Randomized clinical trial, Animal study	41 rats	RAS targeting AHM use after stroke helped preserve cognitive function in hypertensive rats

**Table 6:** Studies that dealt with comparisons of various antihypertensive medications CCB: calcium channel blockers, ARB: angiotensin II receptor blockers, RAS: renin-angiotensin system, AHM: antihypertensive medications, ACEi: angiotensin-converting enzyme inhibitors, VCI: vascular cognitive impairment, VD: vascular dementia, cACEi: centrally acting angiotensin-converting enzyme inhibitors, AD: Alzheimer’s disease, ARB: angiotensin receptor blockers

Authors	Year	Type of study	Total number of patients	Relevant results and conclusions
van Middelaar et al [36].	2017	Randomized clinical trial	3526	Both uses of CCB and ARB are independently associated with a decreased risk of dementia in older people.
Zhuang et al. [37]	2016	Meta-analysis	54,678	RAS targeting AHM, specifically ACEi, might reduce the incidence of VCI and VD.
Zhuang et al. [38]	2016	Meta-analysis	1,268,156	RAS targeting AHM medications might reduce the incidence of dementia. cACEi might prevent cognitive decline.
Zhuang et al. [39]	2016	Meta-analysis	887,205	RAS targeting AHM medications might prevent AD. ARB might reduce the incidence of cognitive impairment of aging.
Peters et al. [40]	2020	Meta-analysis	56,866	Patient aged >65 years: only diuretics showed possible benefit in some analysis Patients aged 65 years: limited data Findings allow clinical freedom to select AHM

Discussion

Cognition and Cardiovascular Risk Factors

One way to ascertain a link between cognitive dysfunction and hypertension can be to study the risk factors of cognitive impairment (CI) (Table 3). Xue et al. looked for factors that could predict the chance of reversion from MCI to normal cognition (NC) [20]. They looked at a total of 17 studies and performed a meta-analysis accordingly. The analysis showed that of the five articles related to the APOEe4 gene, the APOEe4 allele's absence had an association with reversion from MCI to NC. A similar trend was noticed for having no hypertension, no stroke, which led to a greater chance of reversion [20]. This might suggest that dementia unrelated to cardiovascular causes or Alzheimer's disease has a greater chance of recovery. Conversely, hypertension might be a risk factor and a modifiable one at that.

However, the authors admitted limitations to the studies, including variability in study settings and a low number of studies included in the meta-analysis. The study could not stratify based on etiology, and their meta-analysis may have included dementia due to overlapping etiologies [20]. This is a relevant finding, especially when looking at the relationship between AHM and stroke. Xie et al. suggested that AHM can prevent strokes, and preventing strokes can help prevent cognitive dysfunction. They conducted a meta-analysis of 27 RCTs, including hypertensive patients, but not heart failure patients. Their findings showed that ACEi reduced all secondary outcomes, i.e., all-cause mortality, MI, and stroke and that CCB and diuretics significantly decreased the risk of stroke. Since stroke is a risk factor for CI, reducing its risk may help prevent cognitive dysfunction [20,21].

CI, due to hypertension, might not just be limited to a chronic cause. You et al. studied CI in intracerebral hemorrhagic (ICH) patients [22]. They suggested that the systolic blood pressure elevation in the first 24 hours after ICH might be linked to CI. Sun et al. also looked at an acute rise in blood pressure [23]. They conducted an RCT that studied the effect of nicardipine (CCB), nitroglycerin, esmolol (BB), in preventing postoperative cognitive dysfunction (POCD) in elderly patients who underwent surgery and anesthesia, and the incidence of POCD. They found that the patients who received either nicardipine alone or in combination with esmolol had a far lesser incidence of POCD than those that did not receive these interventions. They also specifically mentioned that the absence of ICH complications in all groups, suggesting that POCD may occur independently of complications [23].

The question now arises, how could hypertension, whether acute or chronic, affect cognition? Scharf et al. suggest that hypertension may be asserting its effects by creating changes on a neuronal level [24]. They studied white matter hyperintensity (WMH) via MRI imaging. In their study, they found that WMH, i.e., white matter lesions, were strongly associated with hypertension [24]. Interestingly, they also mentioned that measuring brachial blood pressure might not accurately reflect the effect of atherosclerotic aortic pressure on brain vasculature.

De Heus et al. suggested that fluctuations in BP or blood pressure variability (BPV) could also play a role in developing cognitive disorders (Table 3) [25]. They specifically looked at patients with Alzheimer's disease and found that patients with greater visit-to-visit blood pressure variability had greater CD during the 1.0 and 1.5 years follow up. The authors suggested that a possible cause of Alzheimer's disease in patients with high BPV might involve an impaired clearing of Amyloid β plaques and loss of neuronal function due to damage to cerebral microcirculation from the high blood flow pulsatility [25,41]. They further suggested that this was mainly associated with diastolic BPV as it was linked to a sympathetic drive or endothelial dysfunction. They also suggested that systolic BPV was linked to arterial stiffness [25,42,43]. This can warrant greater research, especially in elderly hypertensive patients, as arterial stiffness increases with age and might be more prevalent in the elderly population.

Another way of looking for a link between hypertension and cognition can be to study the effects of chronic hypertension. Georgakis et al. studied the association of left ventricular hypertrophy (LVH) with CI (Table 3) [26]. The results showed an increased CI risk in patients with LVH in both hypertensive and normal blood pressure patients. The finding was evaluated using electrocardiogram and transthoracic echocardiogram. The authors suggested that the findings could not differentiate between CI that accompanies dementia, Alzheimer's disease, and vascular diseases [26]. Thus, they recommended further research differentiating between these pathologies.

Antihypertensive Medications and Cognition

Many studies have actively looked for a potential link between AHM and cognition; some recent studies have been listed (Table 4). Parsons et al. conducted a meta-analysis and found that AHM significantly reduced stroke [27]. However, they did not have a significant impact on reducing dementia or CD. The authors mentioned that the average follow-up for the studies they included was about 3.1 to 3.5 years. Therefore, they may need to include or conduct studies with a longer follow-up to show the impact on dementia and CD [27]. Hughes et al. did a meta-analysis of only RCTs, and their findings suggested that treating hypertension might decrease dementia and CI incidence [28]. They had a slightly greater duration of follow up, which was reported to be 4.1 years. However, they do note caution, as some of their confidence intervals included one [28].

Similarly, Xu et al. also investigated the AHM role, CD, and dementia [29]. They looked at 10 prospective cohort studies and performed a meta-analysis to look for outcomes such as dementia, AD, CI, and CD. No link was seen between AHM and AD, CI, or CD, but an improvement in the incidence of dementia was seen. This was an interesting finding, and the authors suggested that it might have to do with the type of studies used in the meta-analysis. They mentioned that some of their included studies had inconsistent results [29].

Once it can be established that AHM might help prevent CI, it becomes crucial to understand what the treatment goal should be, and at what age can the AHM exert their greatest effect. SPRINT MIND Investigators for the SPRINT Research Group et al. conducted a study looking at intensive versus standard blood pressure control and its effect on dementia incidence [12]. They randomly assigned the control group with a blood pressure treatment goal of 120 mmHg and the treatment group with a blood pressure treatment goal of 140 mmHg. During follow-up, participants completed cognitive assessments to assess their cognitive status [12].

Results showed that the intensive blood pressure control treatment group had a significantly lower rate of MCI or probable dementia than the control group. The authors admitted that MCI was not the primary outcome of the trial. Though there is a link between MCI and dementia, MCI may also revert to NC. As such, the authors could not conclude whether an SBP goal of less than 120 mmHg is better than a goal of less than 140 mmHg to prevent the risk of dementia [12]. This remains to be further researched. This topic is worth investigating, as an optimal treatment goal could lead to a greater reduction in CI incidence.

As mentioned before, there is an ongoing conversation about what treatment goals should be utilized in elderly hypertensive patients [2,3]. Vazirinejad et al. conducted a clinical trial with 248 participants and measured mini-mental state examination (MMSE) before and after patients underwent hypertension treatment for three months (Table 4) [30]. Their findings showed that the use of AHM in ages greater than 40 significantly improved their cognition performance compared to in ages less than 40 [30]. This showed the importance of treating hypertension optimally, even in the elderly population, as they stand to benefit significantly.

A study done by Moonen et al. allowed us to see a different approach to investigation, as it gave us a view of what might happen to a patient's cognition when AHM are discontinued (Table 4) [31]. Interestingly, low blood pressure in elderly patients is also associated with CD [31,44,14]. When Moonen et al. stopped AHM in the intervention group, they found that the intervention groups increased BP compared to the control group. However, there was no improvement in their cognition. This showed that, on the one hand, the increasing BP did not improve cognition; however, it also didn't worsen it. The authors also mentioned that they had a short follow-up. They could not assess the long-term effect of discontinuing AHM in elderly hypertensive patients [31]. More longitudinal research can be done to explore optimal treatment goals in this patient population.

Specific Antihypertensive Medication Classes and Cognition

Several papers studied individual antihypertensive classes or medications and their effects on cognition (Table 5). Tully et al. point out diuretics as the main drug of use for elderly hypertension patients [32,45]. They did a meta-analysis, which included both RCTs and observational studies and showed that diuretics reduced dementia and AD risk. However, the authors noted the lack of studies done assessing the benefits of diuretics despite being a commonly used AHM. Interestingly, they also stratified the risk reduction by type and mention a reduction of dementia risk by 6% in thiazides, 14% in loop, and 30% in potassium-sparing diuretics [32].

Lawlor et al. and de Jong et al. conducted randomized clinical trials involving Nilvadipine, a dihydropyridine CCB [33,34]. Nilvadipine has previously been shown to lower amyloid-β in animal studies [33]. Lawlor et al. looked at Alzheimer's disease patients and conducted an 18-month RCT by administering nilvadipine in participants with diagnosed mild to moderate stage AD [33]. They excluded anyone with established dementia from other causes. However, they suggested that they could not exclude all other etiologies, as they did not check for brain amyloid biomarkers. The outcome was assessed using the AD assessment scale cognitive subscale-12 (ADAS-Cog12), clinical dementia rating scale sum of boxes (CDR-sb), and disability assessment for dementia (DAD) measurement scales [33].

Their findings showed that nilvadipine had no statistically significant benefit. It can be argued that AD's pathophysiology is different from vascular dementia and stroke; therefore, this finding may show the benefit of CCB is not generalizable to all pathologies of dementia. The authors mentioned a drop of 5 mmHg median in systolic BP during the treatment, which can favor this argument. The authors also mention that if perhaps cerebral hypoperfusion worsened amyloid deposition, early-stage interventions in AD patients with cerebral blood flow (CBF) altering medications can help prevent dementia in AD patients too [33], which has also been discussed elsewhere [25,41].

De Jong et al. also studied the effect of nilvadipine in mild to moderate AD patients [34]. They specifically studied whether a six-month treatment duration would bring out any CBF changes by using a special imaging technique, magnetic resonance arterial spin labeling, or ASL, to look at specific brain regions. Their findings showed that nilvadipine therapy led to greater cerebral blood flow in the bilateral hippocampal area. Global and posterior cingulate cortex blood flow did not change [34]. They mentioned that nilvadipine had around 10 mmHg drop in SBP and that global and regional CBF was stable despite this. They suggested that the increased CBF in the hippocampus was a result of the BP-lowering mechanism. Hypertension may be causing microvascular pathology in AD patients, which was relieved by the use of AHM. However, a short follow-up duration was noted, as well, no measurements on the structural brain and cognitive measure could be done [34].

Ahmed et al. studied CI in the setting of experimental stroke, and specifically looked at the benefit of the renin-angiotensin system (RAS) targeting AHM can provide [35]. They conducted an animal study involving spontaneously hypertensive rats (SHRs) and induced a temporary 60-min stroke experimentally in the middle cerebral artery. After the reperfusion, they treated the SHRs with candesartan, compound 21, or saline. After seven days, some of the saline group received treatment as well [35]. CI was measured using a combination of sensorimotor/neurobehavioral/cognitive-function tests, histopathology, amyloid-β, etc.

Their findings suggested that SHRs given candesartan had less CI incidence after induced stroke versus SHRs given compound 21 or saline [35]. They further recommended the use of compound 21, an agonist of angiotensin II type two receptors, an experimental compound used for in-vitro and in-vivo studies with rats, to possibly decrease the incidence of CI. They found that CI could be prevented even if the treatment was delayed for seven days, suggesting the importance of treating BP effectively after stroke to prevent CI [35]. These few studies show that all classes of antihypertensive medications are associated with a reduced risk of cognitive impairment. However, the effect may be due to AHM in general and not a specific AHM class.

Comparing Antihypertensive Medications Classes

Though many AHM classes have individually shown to decrease the risk of dementia, CI or CD, there are a few studies done that have compared the risk reduction of dementia across classes of AHM (Table 6). van Middelar et al. conducted an RCT with 3,526 participants, with a mean age of 74.4 years, and out of which 1,951 were hypertensive [36]. They looked at the incidence of all-cause dementia, diagnosed using criteria from the Diagnostic and Statistical Manual of Mental Disorders, Fourth Edition (DSM-IV), and used MMSE for evaluation. They had six to eight years follow-up and found that of the patients using AHM at baseline and onwards, 136 participants developed dementia. The authors noted that CCB and ARB had an independent effect on reducing the risk of dementia. They also suggested that this association was strongest in CCB, especially in patients without a history of cardiovascular disease or uncontrolled hypertension. Perhaps this could be linked to the presence of fewer vascular lesions in these patients [36]. This also points towards the importance of starting and maintaining an optimal antihypertensive medication regimen to prevent CD.

**Figure 2:**
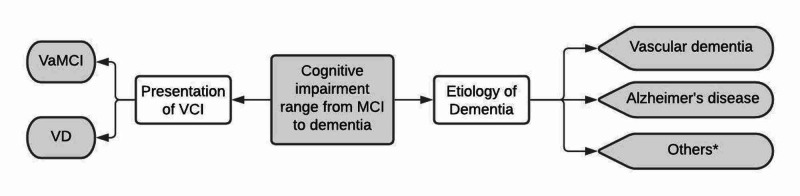
Overview of different etiologies of dementia and the range of presentation of vascular cognitive impairment *Other causes include frontotemporal dementia, Lewy body dementia, pseudodementia, HIV-associated dementia, etc. MCI: mild cognitive impairment, VCI: vascular cognitive impairment, VaMCI: vascular mild cognitive impairment, VD: vascular dementia

Zhuang et al. conducted a series of meta-analyses to look at vascular dementia and AD [37-39]. They believed that RAS targeting AHM may have a greater benefit than others in reducing CD and dementia [39,46,47]. Their findings suggested that RAS blocking AHM might reduce the incidence of dementia [38], vascular cognitive impairment (VCI), and vascular dementia (VD) [37], as well as AD [39]. They also specifically mentioned that ACEi might reduce the incidence of VCI and VD [37]; centrally acting ACEi might prevent CD [38], and ARB might reduce the incidence of cognitive impairment of aging [39]. These different meta-analyses can help us better understand the various presentation of VCI and the etiology and pathophysiology of dementia and the role AHM can play to prevent CI (Figure 2) [37-39]. Blood pressure has been linked to Alzheimer's disease [25]. Individuals with Alzheimer's disease commonly exhibit vascular damage features combined with β-amyloid and tau neuropathology [12,48-50]. These meta-analyses also showed that apart from CCB [33,34], the RAS targeting AHM class might also affect neuropathology.

Peters et al. recently conducted a meta-analysis comparing various classes of AHM [40]. Their study was different because it measured incident CD using a reliable change index (RCI), which standardized a decline in cognition across various tests. Their findings suggested that the use of diuretics in ages greater than 65 led to reduced dementia and CD risk. However, the effect was non-consistent, and they suggested that there could be clinical freedom to choose any antihypertensive class for the treatment of hypertension [40]. These findings also pointed towards conducting studies that more closely study diuretics and their effect on cognition [32,40].

Future Approach

Peters et al. have a different approach by using RCI as most of the studies conducted incorporated different modes of measurements (Table 7). It could be useful to have a standard measurement across studies when investigating changes in cognition. This can be challenging given the variability; however, having various baseline measurements in control groups can help. This will further prevent bias and will lead to more meaningful results.

**Table 7:** Available characteristics of meta-analyses that studied the association between antihypertensive medications and cognition RCT: randomized clinical trials, MMSE: mini-mental status examination, TIA: transient ischemic attack, CD: cognitive decline, AD: Alzheimer’s disease, CI: cognitive impairment, PMH: past medical history, DM: diabetes mellitus, CVD: cardiovascular diseases, VD: vascular dementia, DSM-3R: Diagnostic and Statistical Manual of Mental Disorders Third Edition Revised, DSM-4: Diagnostic and Statistical Manual of Mental Disorders Fourth Edition, NINCDS-ADRDA: National Institute of Neurological and Communicative Disorders and Stroke and the Alzheimer's Disease and Related Disorders Association, NINDS-AIREN: International Workshop of the National Institute of Neurological Disorders and Stroke (NINDS) and the Association Internationale pour la Recherche et l’Enseignement en Neurosciences (AIREN), ICD-9: International Classification of Diseases 9, ICD-10: International Classification of Diseases 10, OXMIS: Oxford Medical Information System, ADL: activities of daily living, CASI: Cognitive Abilities Screening Instrument, MCI: mild cognitive impairment, CDR: Clinical Dementia Rating scale, VCI: vascular cognitive impairment Note: Data were collected by the specific mentions the studies made, and not by evaluating the studies included in the meta-analyses.

Authors	Type of studies included	Mean, median, minimum, or range of age of the patient population (years)	Mean, median, minimum, or range of follow-up time (years)	Mode of measurement used to assess cognition	Etiology present in the patient population
Parsons et al. [27]	14 RCT	>65	>2	Diagnostic criteria for dementia, changes in cognitive test scores (e.g. MMSE)	Stroke, TIA, dementia, CD
Hughes et al. [28]	14 RCT	Mean 69	Mean 4.1	Incidence of dementia (DSM-3R, DSM-4, ICD-10, investigator reported, specialist confirmed, changes in cognitive test scores (e.g. MMSE)	Dementia
Xu et al. [29]	10 prospective cohort study	68.7 to 83.0	2.2–32.0	Incidence of dementia, AD, CI, CD	Dementia, AD, CI, CD (PMH of stroke, DM, CVD)
Tully et al. [32]	15 prospective studies (incl. RCT, longitudinal cohort, database registry)	Median 76.1	Median 6.1	Incident dementia and probable dementia, probable AD (standardized criteria, and the diagnosis made by clinician including DSM-3R, DSM-4, NINCDS-ADRDA, NINDS-AIREN)	Dementia (included AD, VD, mixed dementia, unspecified, and other)
Zhuang et al. [37]	5 studies (3 RCTs, 2 case-control)	-	-	Incidence of VCI (DSM-4, MMSE ≤24, MMSE ≤23, OXMIS, records)	VD, probable VD, CI
Zhuang et al. [38]	10 studies (5 RCT, 3 cohorts, 2 case-control)	-	-	Incidence of dementia, CD (various studies included DSM-4, MMSE, ICD-9, ICD-10, NINCDS-ADRDA, OXMIS as criteria)	Dementia (PMH incl. DM, stroke)
Zhuang et al. [39]	10 studies (1 RCT, 7 cohorts, 2 case-control)	-	3-8	DSM-4, MMSE, ADL, ICD-9, ICD-10, NINCDS-ADRDA, OXMIS, CASI<74 as outcome measures	AD, probable or possible AD, CI, MCI
Peters et al. [40]	27 studies (21 observational cohort studies, 2 clinical trials, 4 RCT)	57.0-93.0 (majority in 70-79). Data was stratified by age, midlife (≤65) or late (>65)	≥1	CD assessed using MMSE screening test. Incident dementia (DSM-3R, DSM-4, CDR score ≥1, international diagnostic evaluation, neuropsychological testing)	Dementia, CD. (No prevalent dementia at baseline)

Another difference in the studies was the pathophysiology of dementia itself. Some studies specifically included patients with a particular etiology of dementia, whereas others were more lenient. From some of the findings, it seems that AHM could affect outcomes in stroke or vascular dementia and other pathologies. This might also help better understand the role of cerebral perfusion and blood pressure in cognitive disorders. However, all these concerns make it difficult to study vascular dementia or cognitive dysfunction in hypertensive patients.

Based on the current studies, it is impossible to say with absolute confidence that one AHM class is more beneficial than others in preventing vascular dementia from chronic hypertension. However, CCB and ARB seem to have shown great benefit. Further research should be geared towards replicating previous clinical studies, creating standard screening measurement scales for elderly hypertensive patients more vulnerable to future cognitive dysfunction, and finding optimal hypertension treatment goals in midlife vs. elderly hypertensive patients. Since dementia or cognitive impairment may develop over time, there is also a need to include or perform studies with a much longer follow up.

Limitations

The analysis of the most current studies done on hypertension and cognition revealed some limitations. One issue was the difference in the type of studies utilized in meta-analysis. Many meta-analyses differed in whether they included prospective cohort studies and clinical trials (Table 7). Xu et al mentioned this mixture of types of studies involved as a limitation [29]. There is also a dilemma of whether to assess the incidence of dementia and other cognitive pathologies as they present and whether their findings can correlate well with studies that looked at genetic markers or imaging techniques such as fMRI or ASL. This might create smaller pools within the meta-analysis studies and possibly lead to greater bias. Another thing would be to carefully select studies because the type, year, and the number of related publications included during that year might lead to bias. Diuretics AHM especially seemed to lack in the number of studies conducted [32,40].

This was not a systematic review; therefore, the quality of the papers studied cannot be ascertained. However, the inclusion and exclusion criteria only used meta-analysis, RCTs, and other clinical trials to limit bias and possibly assess causation. Not all clinical trials were randomized and may not include control groups. One animal study was also included.

## Conclusions

This review looked at studies that showed how and why hypertension might affect cognition and whether AHM plays a role in preventing cognitive impairment. We included meta-analysis, RCT, and clinical trials. The findings showed that the risk factors that affect cognition include hypertension, stroke, age, BPV, and LVH. The use of AHM was associated with a reduced risk of stroke, dementia, CI to various degrees. Studies showed that AHM might be useful across dementia pathologies such as AD and VD. Many AHM classes were individually associated with reducing the risk of cognitive impairment; however, comparing the AHM class effect on cognition was difficult.

We looked at the limitations of studies that included type, number, or mode of measurement of cognition used in meta-analysis. The studies did not show the superiority of any one AHM class in preventing cognition impairment. It might be more beneficial to further research hypertension treatment goals to prevent cognitive impairment in both acute and chronic high BP. It might be useful to measure BP in-office visits, as well as monitor fluctuations in BP, and assess SBP and DBP individually, to better predict the risk of vascular dementia. This review analyzed the trend of recent studies done in studying AHM and cognition.
